# Antimicrobial Protein and Peptide Concentrations and Activity in Human Breast Milk Consumed by Preterm Infants at Risk of Late-Onset Neonatal Sepsis

**DOI:** 10.1371/journal.pone.0117038

**Published:** 2015-02-02

**Authors:** Stephanie Trend, Tobias Strunk, Julie Hibbert, Chooi Heen Kok, Guicheng Zhang, Dorota A. Doherty, Peter Richmond, David Burgner, Karen Simmer, Donald J. Davidson, Andrew J. Currie

**Affiliations:** 1 Centre for Neonatal Research and Education, University of Western Australia, Perth, Western Australia, Australia; 2 School of Paediatrics and Child Health, University of Western Australia, Perth, Western Australia, Australia; 3 Neonatal Clinical Care Unit, King Edward Memorial Hospital for Women, Perth, Western Australia, Australia; 4 School of Public Health, Curtin University, Perth, Australia; 5 School of Women’s and Infants’ Health, University of Western Australia, Perth, Australia; 6 Murdoch Childrens Research Institute, Parkville, Victoria, Australia; 7 University of Melbourne, Melbourne, Victoria, Australia; 8 The University of Edinburgh/MRC Centre for Inflammation Research, Queen’s Medical Research Institute, Edinburgh, United Kingdom; 9 School of Veterinary and Life Sciences, Murdoch University, Perth, Western Australia, Australia; University Hospital Schleswig-Holstein, Campus Kiel, GERMANY

## Abstract

**Objective:**

We investigated the levels and antimicrobial activity of antimicrobial proteins and peptides (AMPs) in breast milk consumed by preterm infants, and whether deficiencies of these factors were associated with late-onset neonatal sepsis (LOS), a bacterial infection that frequently occurs in preterm infants in the neonatal period.

**Study design:**

Breast milk from mothers of preterm infants (≤32 weeks gestation) was collected on days 7 (n = 88) and 21 (n = 77) postpartum. Concentrations of lactoferrin, LL-37, beta-defensins 1 and 2, and alpha-defensin 5 were measured by enzyme-linked immunosorbent assay. The antimicrobial activity of breast milk samples against *Staphylococcus epidermidis*, *Staphylococcus aureus*, *Escherichia coli*, and *Streptococcus agalactiae* was compared to the activity of infant formula, alone or supplemented with physiological levels of AMPs. Samples of breast milk fed to infants with and without subsequent LOS were compared for levels of AMPs and inhibition of bacterial growth.

**Results:**

Levels of most AMPs and antibacterial activity in preterm breast milk were higher at day 7 than at day 21. Lactoferrin was the only AMP that limited pathogen growth >50% when added to formula at a concentration equivalent to that present in breast milk. Levels of AMPs were similar in the breast milk fed to infants with and without LOS, however, infants who developed LOS consumed significantly less breast milk and lower doses of milk AMPs than those who were free from LOS.

**Conclusions:**

The concentrations of lactoferrin and defensins in preterm breast milk have antimicrobial activity against common neonatal pathogens.

## Introduction

One quarter of very preterm infants (<32 weeks gestational age (GA)) develop late-onset sepsis (LOS), most commonly with coagulase-negative Staphylococci (CoNS), 7–14 days postpartum [1, 2]. Gastrointestinal bacterial overgrowth and dysbiosis, as well as poor integrity of the gastrointestinal epithelium, may facilitate translocation of LOS-causing organisms into the bloodstream [3, 4]. Breast milk reduces gastrointestinal bacterial load and translocation in animal studies [5], lowers intestinal permeability in humans [6], and importantly, reduces the incidence of LOS in preterm infants [7]. Nevertheless, the protective mechanisms involved are unclear.

Antimicrobial peptides (also known as cationic host defence peptides) and proteins (AMPs) are present in many secretions including human breast milk [8]. These molecules have broad-spectrum antimicrobial activity *in vitro* against bacteria, viruses, and fungi, as well as synergistic activity with conventional antibiotics [8–11]. Human milk and the purified AMPs that it may contain have reported *in vitro* bacteriostatic or bactericidal activity against neonatal pathogens [12–14]. In addition, many AMPs have important modulatory properties in inflammation and immunity [15], and may alter the gut microbiome [16]. However, there is a paucity of data available on the concentrations of AMPs in preterm breast milk. Lactoferrin (LF) and lysozyme concentrations in preterm milk have been reported, but there are conflicting data on levels in preterm, as compared to term, breast milk [17–19]. The effects of the levels and resulting activity of AMPs in human milk against neonatal pathogens, particularly in the uniquely vulnerable preterm population, have not been established.

The aim of this study was to quantify the antimicrobial activity of prototypical AMPs in breast milk from preterm infants’ mothers. We hypothesised that milk AMPs are present in sufficient quantities to inhibit LOS-causing bacterial growth and that relative deficiencies in breast milk AMPs concentrations and antibacterial functions would be associated with increased incidence of LOS in very preterm infants. Five AMPs were quantified and the activities of four of these present in the majority of breast milk samples were investigated.

## Methods

### Study participants

This study was approved by the institutional Ethics Committee at King Edward Memorial Hospital, Perth, Western Australia. Written informed consent from mothers giving birth at ≤32 weeks gestation was obtained from participants before collection of clinical data and samples. Mothers of infants with major congenital malformations, chromosomal abnormalities, and individuals with insufficient understanding of English to give consent were excluded from the study. Clinical data were collected on all participating infants from birth to 28 days postpartum. Placentae were examined for evidence of histological chorioamnionitis by an experienced perinatal pathologist blinded to all other clinical details, using the method of Redline [20]. Ninety-six mothers provided breast milk samples on days 7 and/or day 21 (70 mothers provided both samples). The demographics of these 96 mothers and their infants (n = 107) are shown in Table 1.

**Table 1:** Characteristics of study cohort.

**Characteristics of study cohort**	**Value**	**Range**
**Maternal**		
Caesarean section^a^	60 (62.5)	
Preterm premature rupture of membranes^a^	57 (59.4)	
Antenatal steroids^a^	94 (97.9)	
Antibiotics during labour^a^	55 (57.3)	
**Neonatal**		
Gestational age^b^ (weeks)	27.24±2.0	22.85–32.43
Birth weight^c^ (grams)	930	455–1816
Early-onset sepsis^a^ (EOS)	3 (2.8)	
Late-onset sepsis^a^ (LOS)	24 (22.4)	
Histological chorioamnionitis^a^	Yes	42 (39.3)
	No	44 (41.1)
	Not available	21 (19.6)

Clinical characteristics of the mothers (n = 96) and neonates (n = 107) from the preterm cohort. Statistical values given:

^a^n (%);

^b^mean±SD;

^c^median.

### Milk collection and processing

All preterm mothers in the study were educated by clinical staff on hygienic collection and storage of expressed breast milk, according to clinical protocols. Breast milk was expressed by mothers into sterile containers and transported in an insulated container to the neonatal intensive care unit (NICU) milk room with an ice brick. Milk was only accepted by the NICU if the sample had been stored either 4°C for <48 hours, or RT for <4 hours. Once received, expressed milk was preferentially stored in the NICU at -20°C and thawed for consumption when required, unless no frozen milk was available, in which case refrigerated milk was used for feeding preterm infants. Research samples were collected when milk was prepared for consumption by preterm infants on days 7±2 and 21±2 postpartum, prior to any fortification of milk, in order to minimise interventions to normal milk storage and feeding protocols. Individual sample storage details were not collected.

Research samples of ≤5 mL of breast milk were transported to the laboratory on ice and maternal cells pelleted and removed by centrifugation at 500 x *g* for 5 min. Milk supernatant was collected and frozen at -80°C for batch analyses. In order to remove any milk-resident bacteria, thawed milk samples were skimmed three times by centrifugation at 6,000 x *g* for 10 min, and the liquid fraction collected. Preliminary experiments demonstrated that a median of 3.4 × 10^4^ CFU/mL (range <1 × 10^2^ to 4.1 × 10^6^ CFU/mL) of aerobic milk-resident bacteria were detected in milk samples, and that 99.6% of the bacterial load could be removed through this process.

### AMP quantitation by enzyme-linked immunosorbent assay (ELISA)

We selected AMPs to test based on the following criteria: AMPs reported in human breast milk, whose concentrations in milk are in the reported range of effective concentrations in the literature against LOS bacteria, especially *S. epidermidis*, where a suitable antibody pair could be purchased at the time of assay development. The concentrations of lactoferrin (LF), human beta defensins 1 and 2 (HBD1, HBD2) and human cathelicidin LL-37 were measured in skimmed milk at an appropriate dilution using sandwich ELISAs developed in-house. The antibody pairs used for capture and detection for each ELISA were as follows: mouse IgG_1_ anti-human LF (clone 2B8; ab10110; final concentration 0.5 μg/mL) and biotinylated rabbit polyclonal IgG anti-human LF (ab25811; final concentration 0.5 μg/mL), mouse monoclonal IgG_1_ anti-human HBD1 (clone M11-14b-D10; ab14425; final concentration 0.5 μg/mL) and biotinylated rabbit polyclonal IgG anti-human HBD1 (ab84245; final concentration 0.5 μg/mL), goat polyclonal IgG anti-human HBD2 (ab109570; final concentration 1.0 μg/mL) and biotinylated goat polyclonal IgG anti-human HBD2 (ab83509; final concentration 0.5 μg/mL), and rabbit polyclonal IgG anti-human LL-37 (PA-LL37-100; final concentration 1.0 μg/mL) and biotinylated rabbit polyclonal IgG anti—human LL-37 (PA-LL37BT-100; final concentration 0.5 μg/mL). LF, HBD1 and HBD2 antibodies were purchased from abcam (Cambridge, England) and LL-37 antibodies were purchased from Innovagen (Lund, Sweden).

The level of HD5 was measured using an indirect ELISA. Polyclonal rabbit anti-HD5 antibody (HDEFA51-A; Alpha diagnostics, San Antonio, TX, United States; final concentration 1.0 μg/mL), followed by horseradish peroxidase (HRP)-linked anti-rabbit IgG, (7074; Cell Signaling Technology, Danvers, MA, United States; used at 1 in 500 dilution) was used for detection.

Standard curves from serial dilutions of purified human LF (Aviva systems biology, San Diego, CA, United States), recombinant HBD1, HBD2, or LL-37 (Innovagen), or a control peptide from the active region of human HD5 (US Biological, Salem, MA, United States) were used to interpolate concentrations in samples using a five-parameter logistic (5PL) curve fit. Avidin-HRP (eBioscience, San Diego, CA, United States) was added to biotinylated antibodies for detection. All ELISAs were developed using 3,3’,5,5’– tetramethylbenzidine substrate (eBioscience) and the reaction stopped using 1 M orthophosphoric acid (ChemSupply, Gillman, Australia). Absorbance was measured at 450 nm on a spectrophotometer. Samples below the detection limit were assigned an arbitrary concentration equal to the value of the lower limit of detection of the assay (78 pg/mL for LF, 31 pg/mL for HBD1, 1,563 pg/mL for HBD2, 781 pg/mL for LL-37, and 39 pg/mL for HD5). Intra- and inter-assay variability were calculated from a milk sample designated as the quality control (QC), added in duplicate to each plate. In all ELISA assays, inter-assay variability was <5% and intra-assay variability between replicates was <7%, based on the reproducibility of the QC standard (n = 2–7 plates tested).

### Milk protein measurement

The total protein content in milk samples was quantified using the bicinchoninic acid (BCA) assay (ThermoFisher Scientific, Scoresby, Australia), modified to include a milk sample as a protein standard (kindly provided by Prof. P. Hartmann’s laboratory, University of Western Australia, Australia) with protein content previously determined using the Kjeldahl procedure [21].

### Milk antimicrobial activity

The direct antimicrobial activity of the preterm milk samples against four neonatal bacterial pathogens was assessed after inoculating skimmed breast milk with 1 × 10^6^ CFU/mL of bacteria, and measuring the number of viable bacteria recovered after 4 h of incubation. Relative growth-inhibition capacity of human milk was calculated as the inverse percentage of live bacteria in milk compared to low birth weight infant formula (LBWF; S26 Gold, Wyeth Pty Ltd, Parramatta, Australia, kindly supplied by Dr. G. McLeod, King Edward Memorial Hospital, Australia) that was inoculated with the same bacterial species.

Briefly, cultures of *Escherichia coli* (ATCC11775) in Luria Bertani Broth (PathWest Laboratory Medicine WA Media, Mount Claremont, Australia), or *Staphylococcus epidermidis* (WT1457; an invasive clinical isolate, kindly provided by Dr. Michael Otto, National Institutes of Allergy and Infectious Diseases, MD, USA), *Staphylococcus aureus* (ATCC29213), and *Streptococcus agalactiae* (Group B *Streptococcus*; M141 serotype 1a clinical isolate kindly provided by Prof. Lyn Gilbert, Institute of Clinical Pathology and Medical Research, Sydney, Australia) in heart infusion broth (Oxoid, Hampshire, England), were grown to log phase. Bacteria were washed by centrifugation at 4,000 x *g*, adjusted to 2 × 10^7^ cells/mL in saline, and 2.5 μL of the bacterial preparation was mixed with 47.5 μL of human milk or LBWF in a 96-well polypropylene plate and incubated in a humidified 5% CO_2_ incubator for 4 h at 37°C. After incubation, inoculated milk or LBWF samples were diluted in phosphate buffered saline (PBS; Invitrogen, Mount Waverley, Australia) from 1 × 10^-1^ to 1 × 10^-6^ dilutions, and 10 μL of each dilution was spotted onto one-sixth of an agar plate, spread with a sterile loop, then incubated overnight as previously described.

Selective and differential media (Mannitol Salt agar and MacConkey no. 3 agar from PathWest Media, and GBS agar from Oxoid) were used in all experiments involving milk and LBWF growth controls to ensure that inoculated bacteria and not milk-resident bacteria were detected after incubation. Blood agar was used where selective agar was not required (*i.e.* in LBWF spiking experiments). Comparisons were not made between experimental treatments using different agars. Dilutions of samples that produced between 10–100 detectable bacterial colonies were counted to quantify the colony forming units (CFU/mL) after the incubation.

### Antimicrobial activity of purified AMPs

To assess the direct antimicrobial activity of AMPs at levels detected in breast milk, LBWF was spiked with human milk-derived LF (Athens Research Technology, Athens, GA, United States; lyophilised solution contained 50 mM Tris-HCl with 200 mM NaCl), reconstituted in water and diluted in LBWF to final concentrations of (9.5 mg/mL, 3.8 mg/mL, and 0.5 mg/mL), recombinant HBD1 (Innovagen), reconstituted in PBS containing 0.05% BSA and diluted in LBWF to final concentrations of 1.7 μg/mL, 68 ng/mL, and 1.5 ng/mL, recombinant HBD2 (Innovagen) reconstituted in PBS containing 0.05% BSA and diluted in LBWF to final concentrations of 940 ng/mL, 6 ng/mL, and 0.8 ng/mL, or control HD5 peptide (US Biological) reconstituted in PBS containing 0.05% BSA and diluted in LBWF to final concentrations of 1.1 ng/mL, 130 pg/mL, and 40 pg/mL. The concentrations were derived from the high, median, and low values detected in human milk samples using ELISA. All defensin preparations were added into LBWF at ≤2 parts in 100 from the peptide stocks, but due to the high concentrations of LF and the limitations of its solubility, LF solutions were prepared 25 parts in 100 (high), 10 parts in 100 (median), and 1.3 parts in 100 (low) from a 38 mg/mL solution. In addition to the activity of single AMPs in LBWF, the synergistic activity of AMPs was tested using a combination of the median concentrations of HBD1, HBD2, HD5, and LF in LBWF. Bacterial growth was assessed as described above.

### Antibacterial effects of breast milk and LF in the presence of iron

Four breast milk samples with average bacteriostatic activity against bacterial species and a median LF concentration of 4.3 mg/mL (range 2.52–4.90 mg/mL) were selected to test the effects of iron on bacteriostatic activity of breast milk. Breast milk samples and LBWF were treated with water and LF (human milk derived; final concentration 3.8 mg/mL), water and 1 mM ferric citrate (Sigma-Aldrich, Castle Hill, Australia), or both LF and ferric citrate, each added at a one part in ten dilution to milk, and incubated with bacteria as previously described.

### Late-onset sepsis case-control study

Late-onset sepsis was defined as both a positive blood culture and an elevated blood C-reactive protein (CRP>15 mg/L) detected within 72 h of the positive blood culture. The organisms causing LOS in the study cohort are shown in Table 2. Twenty preterm infants with definite LOS were matched to twenty non-LOS control infants by GA, birth weight, presence/absence of histological chorioamnionitis, mode of delivery, and exposure to antibiotics during labour (Table 3). This sample size was calculated to be sufficient to detect a difference of one standard deviation between the two groups in continuous outcome variables with >80% power at a significance level of α = 0.05. Four LOS cases with a non-septic twin were excluded, since their respective milk could not be assigned to either group.

**Table 2:** Organisms isolated from positive blood cultures in twenty preterm infants with LOS in the case-control study.

**Organisms identified from positive blood cultures**	**Number (%)**
**Total Gram-positive organisms**	**17 (85)**
Total CoNS	17 (85)
• CoNS—not further specified	10 (50)
• *Staphylococcus epidermidis*	5 (25)
• *Staphylococcus haemolyticus*	1 (5)
• *Streptococcus mitis*	1 (5)
**Total Gram-negative organisms**	**6 (30)**
*Bacillus sphaericus*	2 (10)
*Escherichia coli*	2 (10)
*Enterobacter cloacae*	1 (5)
*Enterococcus faecalis*	1 (5)

Four of twenty infants were infected with two species of bacteria.

**Table 3:** Comparison of clinical data for cases and controls used in the nested case-control study of breast milk antimicrobial molecules.

**Clinical characteristics**	**Value**	**LOS cases (n = 20)**	**Non-LOS controls (n = 20)**	**p-value**
Gestational age^a^ (weeks)		26.40±2.187	26.70±1.803	0.204
Birth weight^b^ (g)		740 [646.3–997.5]	817.5 [735–1058]	0.444
Histological chorioamnionitis^c^	Yes	7/20 (35)	8/20 (40)	0.346
	No	11/20 (55)	7/20 (35)	
	Not available	2/20 (10)	5/20 (25)	
Antibiotics during labour^c^	Yes	12/20 (60)	11/20 (55)	0.500
	No	8/20 (40)	9/20 (45)	
Antenatal steroid exposure^c^	Yes	20/20	20/20	1.00
Smoker during pregnancy^c^	Yes	7/20 (35)	3/20 (15)	0.137
Caesarean section^c^	Yes	12/20(60)	13/20 (65)	0.5
Multiple birth^c^	Twins	2/20 (10)	2/20 (10)	0.698
Infant sex^c^	Male	13/20 (65)	9/20 (45)	0.170
Probiotics given ^c^ (infant)		5/20 (25)	6/20 (30)	0.500
Preterm Premature Rupture of membranes^c^		15/20 (75)	12/20 (60)	0.250
**Milk feeding data (days 1–28)**				
Day of first breast milk feed^b^ (MOM and/or PDHM)		5 (4-7)	3 (2-5)	0.084
Postnatal age (days) full enteral feeds reached^b^ (150 mL/kg/d)		28* (23.5–28*)	19.5 (13.5–28*)	**0.028**
Days without enteral feeding^b^		6.5 (3.25–14.25)	4.5 (1–6)	**0.020**
Consumed any PDHM^c^		3/20 (15)	6/20 (30)	0.451
Average breast milk (MOM and/or PDHM) consumed over 28 days^b^ (mL/kg/d)		24.36 (1.163–59.74)	98.88 (37.33–153.1)	**0.001**
Cumulative breast milk dose at day 7 (MOM and/or PDHM)^b^ (mL/kg)		7.69 (1.395–63.62)	26.76 (2.57–220.2)	**0.046**
Supplemented with preterm infant formula^c^		1/20 (5)	1/20 (5)	1.00

Infant n = 20 in each group, however, at day 21, only 14 LOS infants and 17 non-LOS infants mothers’ provided milk. p<0.05 considered significant; shown in bold font.

^a^Mean±SD;

^b^Median (IQR);

^c^Proportion (%);

MOM = mothers’ own milk; PDHM = pasteurised donor human milk.

*Data were only collected on milk feeds to day 28, therefore infants who had not reached full doses by day 28 were designated as receiving milk at day 28 arbitrarily (n = 12 LOS, 5 non-LOS).

The median daily and cumulative doses of combined mothers’ own milk (MOM) and pasteurised donor human milk (PDHM) consumed during the first 28 days postpartum (mL/kg) were calculated for infants in the nested case-control study using the recorded daily volume of milk consumed and the body weight of the infant. Full enteral feeds were defined as a milk volume of 150 mL/kg/d. Time to reach full feeds was recorded up to a maximum of 28 days postpartum, after which the maximum value was arbitrarily assigned to infants who had not reached full feeds by the end of the study period (5 non-LOS controls, 12 LOS cases).

Doses of AMPs consumed by preterm infants on days 7±2 and 21±2 were calculated using the concentration in milk and dose of MOM consumed on that day (mL/kg). Infants fed PDHM (one in each group at day 7, two in each group at day 21) or whose mother did not provide a milk sample on these days (none at day 7, four LOS and one non-LOS at day 21) were excluded from the analysis because AMP concentration was not known for the milk consumed.

### Statistical analysis

Nonparametric tests were used in the analysis of all ELISA and milk consumption data, based on the skewed distribution and Shapiro-Wilk normality tests. Temporal changes to AMP levels in all participants providing samples were detected by comparing day 7 and 21 milk samples from the same individual using a paired Wilcoxon signed-rank test. Spearman’s correlation coefficient (rho; *ρ*) was used to determine if correlation was detected between milk total protein and specific AMP concentrations, or bacteriostatic activity of milk and AMP concentrations. Growth of bacteria in milk was compared to LBWF using Kruskal-Wallis tests with Dunn’s multiple comparison post-tests. The temporal changes to bacteriostatic activity of milk were compared as described for AMP levels. In LBWF spiking experiments, colony-forming units had an approximately log-normal distribution, and log-transformed data were compared using one-way analysis of variance (ANOVA) with Dunnett’s multiple comparison post-tests comparing all treatments to the control column. An unpaired t-test was used for comparisons between the median concentration of lactoferrin spiked into LBWF and the cocktail of AMPs in LBWF. A paired t-test was used to compare breast milk samples before and after spiking with ferric citrate.

In the case-control study, milk consumption data were available for all forty infants throughout the first 28 days postpartum, although some mothers did not provide milk at day 21. Therefore, twenty pairs of day 7 milk samples and eleven pairs (15 LOS, 17 non-LOS samples) of day 21 breast milk samples were compared for experimental results in the case-control study using paired analyses. Given that some infants whose mother did not provide a milk sample were not consuming milk on the days that lactoferrin and HBD1 doses were calculated, this allowed inclusion of zero data for these infants in the dose consumed. In addition, some infants consumed PDHM of unknown AMP composition. Therefore, data from these infants were excluded from AMP dose calculations; this resulted in AMP dose data on nineteen LOS and nineteen non-LOS infants (eighteen pairs) at day 7 and fourteen LOS and seventeen non-LOS infants (eleven pairs only) at day 21. Categorical paired clinical data were compared using Fisher’s exact tests, and paired continuous data were compared using Wilcoxon signed-rank tests. GraphPad software was used to interpolate ELISA concentrations and compare continuous data (GraphPad Prism version 5.00 for Windows, GraphPad Software, San Diego, CA, USA; www.graphpad.com), and SPSS software version 22 (IBM Corp., Armonk, NY, United States; www.ibm.com) was used to compare continuous variables with categorical clinical data. All hypotheses tested were two-sided and p-values <0.05 were considered to be statistically significant, except where appropriate adjustment were made for multiple comparisons.

## Results

### Preterm breast milk AMP and protein concentrations

**Figure 1:**
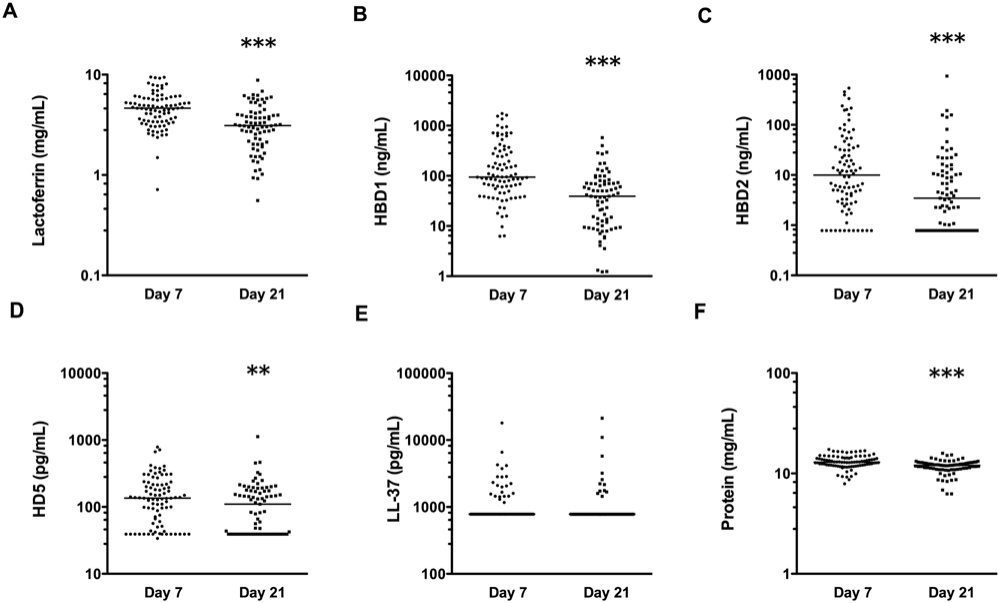
Concentrations of soluble factors measured in breast milk. Concentrations of LF (A); HBD1 (B); HBD2 (C); HD5 (D); LL-37 (E); and total protein (F), measured in day 7 (n = 88) and day 21 (n = 77) breast milk samples using ELISA or BCA assay for protein. All data are shown on a log scale with line at median. **p <0.01, ***p<0.001; comparing levels at 7 and 21 days postpartum using Wilcoxon signed-rank tests.

The median concentrations of all AMPs, except LL-37, were significantly higher on day 7 than day 21 (p<0.01, all comparisons; Fig. 1A–D). The most abundant AMP in the breast milk samples was LF with a median concentration of 4.59 mg/mL on day 7 and 3.13 mg/mL on day 21 (contributing 35.5% and 26% of the total milk protein content, respectively; range 0.56–9.46 mg/mL across all samples). The defensins were found at lower concentrations; median concentration of HBD1 of 94 ng/mL at day 7 and 39 ng/mL at day 21 (range 1.2–1,745 ng/mL across all samples). HBD2 median concentrations were 10 ng/mL and 3.4 ng/mL, respectively. HBD2 levels were below the detectable cut-off in 12 of 88 day 7 samples, and 23 of 77 day 21 samples (a maximum level of 937 ng/mL was detected). HD5 concentrations were a median of 135 pg/mL and 110 pg/mL, respectively. HD5 levels were below the detectable cut-off in 19 out of 88 samples at day 7 and 31 out of 77 samples at day 21 (a maximum level of 1,119 pg/mL was detected). LL-37 levels were below the limit of detection in the majority of breast milk samples (75% on day 7 and 85% on day 21; range 781 pg/mL–21,200 pg/mL; Fig. 1E) therefore we did not include LL-37 in further analyses. For samples with detectable LL-37, the median concentrations were 2,240 and 2,160 pg/mL, respectively.

The total protein concentration in breast milk was between 6.3 and 17.4 mg/ml in all samples (Fig. 1F), and was significantly higher at day 7 than at day 21 (median 12.9 mg/mL versus 12.0 mg/mL; p<0.001). For all day 7 and 21 samples (n = 165), protein concentrations were weakly correlated with LF (*ρ* = 0.31), HBD1 (*ρ* = 0.26), HBD2 (*ρ* = 0.19), HD5 (*ρ* = 0.19) and LL-37 (*ρ* = 0.15) concentrations in milk (all p<0.05). The concentration of LF correlated with the concentrations of HBD1 and HBD2 (*ρ* = 0.35 and *ρ* = 0.18; p<0.05), and levels of HBD1 and HBD2 were significantly correlated (*ρ* = 0.31; p<0.001).

### Breast milk bacteriostatic activity

**Figure 2:**
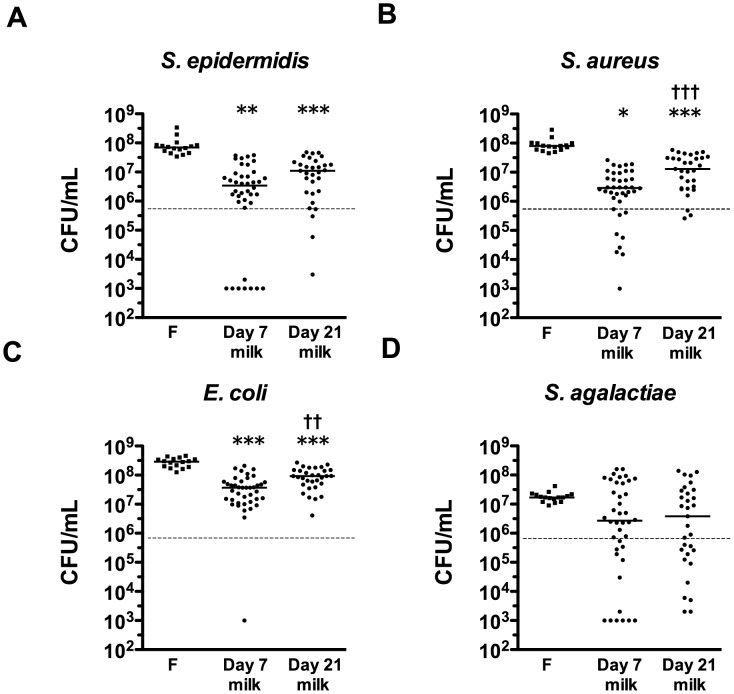
Bacterial growth-inhibition activities of breast milk samples. Colony-forming units of (A) *S. epidermidis*; (B) *S. aureus*; (C) *E. coli*; or (D) *S. agalactiae*, after 4 h incubation in either LBWF (F; n = 16), day 7 (n = 40) or day 21 (n = 31) skimmed preterm breast milk samples from participants in the case-control study. The dashed line shows median starting inoculum. Data show individual and median values on a log scale. A value of 10^3^ CFU/mL was assigned to samples where the colony count was below the limit of detection of the assay. Symbols depict the groups where statistically significant comparisons were made (level of significance indicated by multiple symbols; e.g. *p <0.05, **p<0.01, ***p<0.001), comparing growth in LBWF to growth in preterm breast milk samples by ANOVA with Dunn’s multiple comparison test (*) or comparing growth in day 7 and day 21 paired breast milk samples by Wilcoxon signed-rank tests (†).

The inhibitory activity of preterm breast milk against common neonatal pathogens was tested *in vitro* in a subset of mothers included in the case-control study (n = 40 day 7, n = 31 day 21 samples). *S. epidermidis*, *S. aureus*, and *E. coli* grew by approximately two logs in LBWF. This growth was significantly inhibited by breast milk samples (Fig. 2). *S. agalactiae* grew by approximately one log in LBWF, greater growth than observed in two-thirds of the breast milk samples, although this difference was not significant. In comparison to growth in LBWF, day seven breast milk samples were able to inhibit the growth of *S. epidermidis*, *S. aureus* and *E. coli* by a median of 96.0%, 96.6%, and 90.0%, respectively. Day 21 breast milk was less effective at controlling growth, with median inhibition of these bacterial species of 79.0%, 84.6%, and 62.2%, respectively.

The concentration of LF in breast milk showed negative correlation with the colony forming units of *E. coli* and *S. aureus* after incubation with breast milk (*ρ* = -0.24; p = 0.04 and *ρ* = -0.2; p = 0.08, respectively) and HBD1 and HBD2 concentrations were negatively correlated with *E. coli* CFU (*ρ* = -0.59; p<0.001, and *ρ* = -0.36; p<0.01, respectively). Significant correlation between HD5 concentrations in breast milk and viability of other bacterial species was not detected.

### Bacteriostatic activity of AMP levels in infant formula

**Figure 3:**
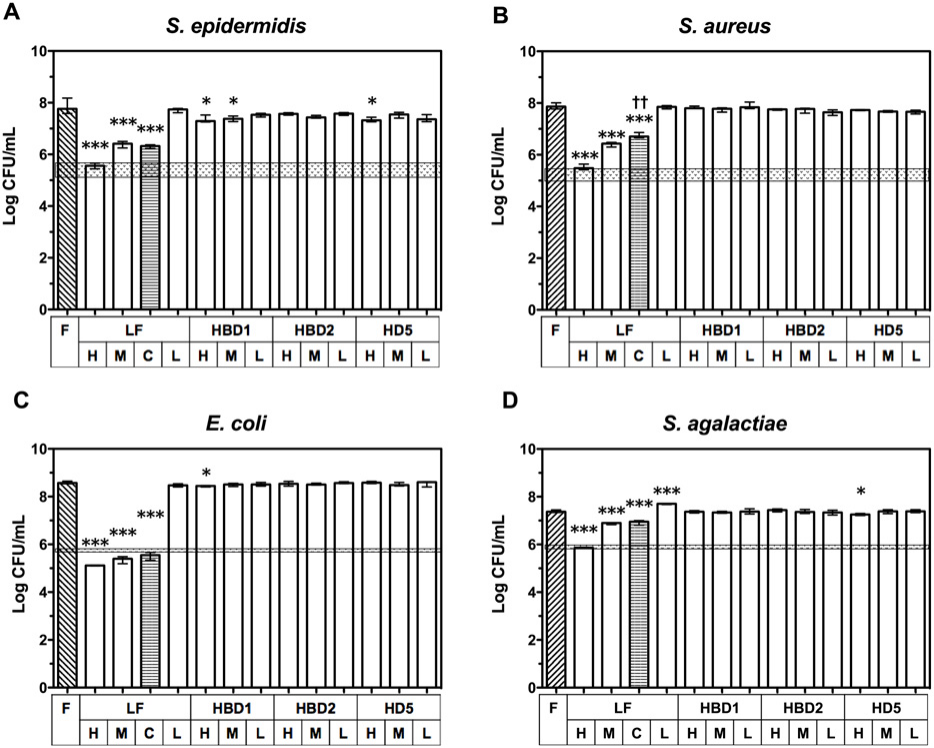
Inhibition of bacterial growth in LBWF spiked with antimicrobial proteins and peptides (AMPs). Results from two experiments show median log-transformed colony-forming units (±interquartile range; n = 4) on a log scale of remaining CFU/mL from an inoculum of ~1 × 10^6^ of: (A) *S. epidermidis*; (B) *S. aureus*; (C) *E. coli*; or (D) *S. agalactiae* after 4 h incubation in LBWF (diagonal lined pattern) spiked with either no AMP (F; n = 8 shows both controls combined, statistical tests were performed using values from relevant day only), or with the low (L), median (M) or high (H) concentration of LF, HBD1, HBD2 or HD5 detected in breast milk samples. C = cocktail of LF, HBD1, HBD2 and HD5 at median concentrations (horizontal lined pattern). Shaded area indicates range of starting inoculum. Symbols depict the groups where statistically significant comparisons were made on log-transformed data (level of significance indicated by multiple symbols; *p <0.05, **p<0.01, ***p<0.001) in ANOVA with Dunnett’s multiple comparison tests of all high, median and low spiked treatments to LBWF control (*), or in a t-test of median LF to cocktail of LF plus other molecules (†).

After observing antimicrobial activity in the breast milk samples and the correlation with AMP concentrations, we aimed to determine if physiological milk levels of individual AMPs were independently capable of inhibiting bacterial growth in LBWF. The addition of LF to LBWF at doses equivalent to the median concentration measured in preterm breast milk samples (3.8 mg/mL) had >50% bacteriostatic effect against all bacterial species, with >97% inhibition of growth for *S. epidermidis*, *S. aureus* and *E. coli*, and 67% for *S. agalactiae* (Fig. 3). The effect was dose-dependent, with inhibition of all species >97% when 9.5 mg/mL LF (equivalent to the highest concentration detected in preterm breast milk) was used. No significant effect on growth inhibition was seen when 0.5 mg/mL LF (the lowest concentration detected in preterm breast milk) was added to LBWF. In contrast, HBD1 inhibited *S. epidermidis* at high and median concentrations (median inhibition 49% and 38%, respectively; p<0.05), and at the high concentration, HBD1 inhibited *E. coli* growth by 29% (p<0.05). Neither *S. aureus* or *S. agalactiae* were significantly inhibited at any milk concentrations of HBD1. HD5 inhibited 48% of *S. epidermidis* growth and 27% of *S. agalactiae* growth at high concentrations (p<0.05), but had no effect at other concentrations or for other organisms. HBD2 had no inhibitory effects for any organism. Furthermore, a cocktail of median doses of LF, HBD1, HBD2, and HD5 to LBWF did not increase inhibition compared to LF alone and, rather, the cocktail was less effective at inhibiting the growth of *S. aureus* was less effective at inhibiting growth (p<0.01). LL-37 was not included in these experiments due to the poor rate of detection in the breast milk samples.

### Bacteriostatic activity of LF and milk in the presence of exogenous iron

**Figure 4:**
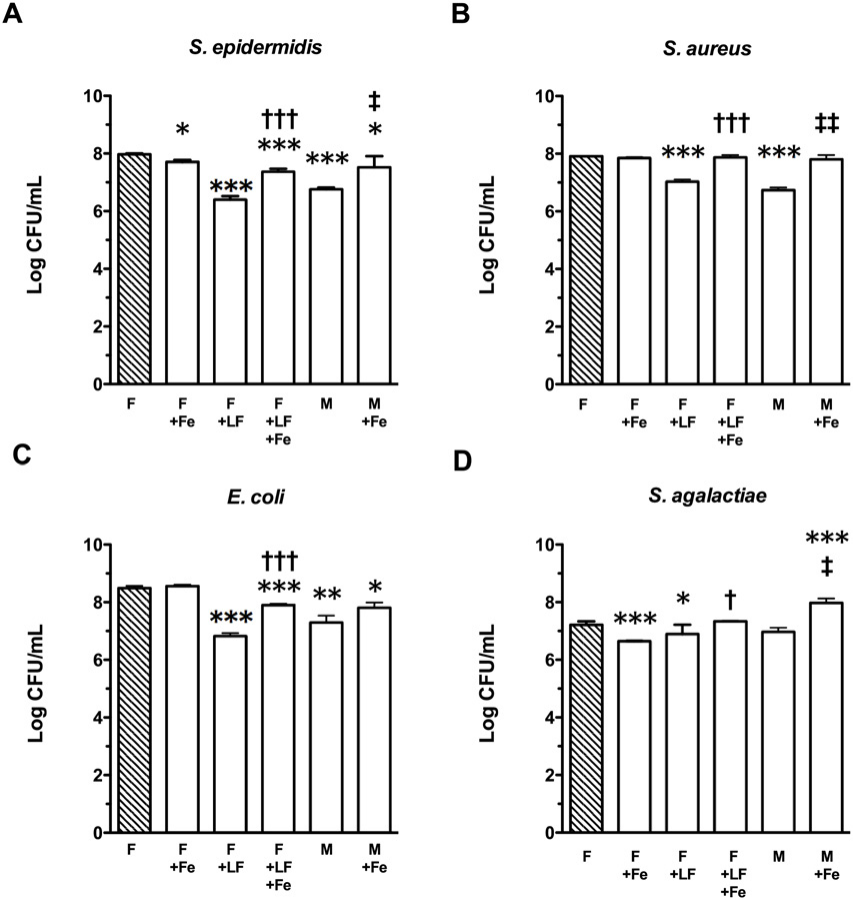
Iron-dependence of inhibitory activity of lactoferrin-spiked LBWF or breast milk against LOS pathogens. Bars show log-transformed medians and interquartile ranges from four replicates of bacterial colonies (Log CFU/mL) of (A) *S. epidermidis*; (B) *S. aureus*; (C) *E. coli*; or (D) *S. agalactiae*, after 4 h growth in LBWF control (shaded bar(“F”)), or LBWF or skimmed preterm breast milk (“M”) ± lactoferrin (“LF”; 3.8 mg/mL) and/or 1 mM ferric citrate (“Fe”). F control shows combined results of two separate experiments, however, treatment was statistically compared to experimental results on that day only. Symbols depict the groups where statistically significant comparisons were made on log-transformed data (level of significance indicated by multiple symbols; e.g. *p <0.05, **p<0.01, ***p<0.001), where (*) shows treatment compared to LBWF control after Dunnett’s multiple comparison test; (†) shows a significant result from an unpaired t-test comparing lactoferrin spiked LBWF ± ferric citrate; and (‡) shows a significant result from a paired t-test comparing four skimmed breast milks ± ferric citrate.

Ferric iron was added to breast milk or LBWF containing lactoferrin to test the hypothesis that the observed inhibitory effects of lactoferrin against bacteria are iron-dependent. Addition of exogenous iron to LF-spiked LBWF significantly reduced the inhibitory activity of these solutions (Fig. 4). In LF-spiked LBWF, *S. epidermidis* median growth-inhibition compared to LBWF alone decreased from 97% to 70% when ferric citrate was added (p<0.001) and, in breast milk, median growth inhibition of 95% decreased to 64% (p<0.05). In LF-spiked LBWF, addition of iron reduced inhibition of *S. aureus* from 87% to 8% (p<0.001) and, in breast milk, a decrease from median 93% to 10% inhibition was observed (p<0.01). The addition of iron to breast milk or LF-spiked LBWF reduced inhibition of *E. coli* from 94% to 79% (not significant) and 98% to 76% (p<0.001), respectively. For *S. agalactiae*, addition of ferric citrate to LF-spiked LBWF decreased inhibition from 56% to 0% (p<0.05). For breast milk, the addition of ferric citrate led to a change from 38% inhibition to almost five times the growth of *S. agalactiae* observed in untreated LBWF (p<0.05). The addition of ferric citrate to LBWF in the absence of LF did not increase bacterial growth, but inhibited *S. agalactiae* by 79% compared to LBWF alone (p<0.001).

### Breast milk AMP levels and LOS in case-control study

In order to determine if deficiencies in breast milk AMP concentrations or antibacterial activity against LOS pathogens were associated with the development of LOS in preterm infants, we compared the levels of AMPs and bacterial inhibition in a subset of matched day 7 and 21 breast milk samples from mothers of infants with and without confirmed LOS. A trend towards lower concentrations of AMPs and increased growth of *S. epidermidis*, *S. aureus* and *E. coli* in breast milk from LOS mothers was observed, however, differences were not statistically significant at either day 7 or day 21 (S1 and S2 Figs.).

### Consumption of breast milk and maternal AMPs in LOS case-control study

Since there were no intrinsic differences in AMP levels or antimicrobial activity between the breast milk samples from LOS cases and controls, but LF and HBD1 had clear activity against LOS pathogens, we examined whether differences in total breast milk consumption, or the effective total doses of LF or HBD1 consumption by infants may associate with development of LOS. Other AMP doses were not calculated since the concentration was below the limit of detection in several milk samples, and limited activity was observed.

**Figure 5:**
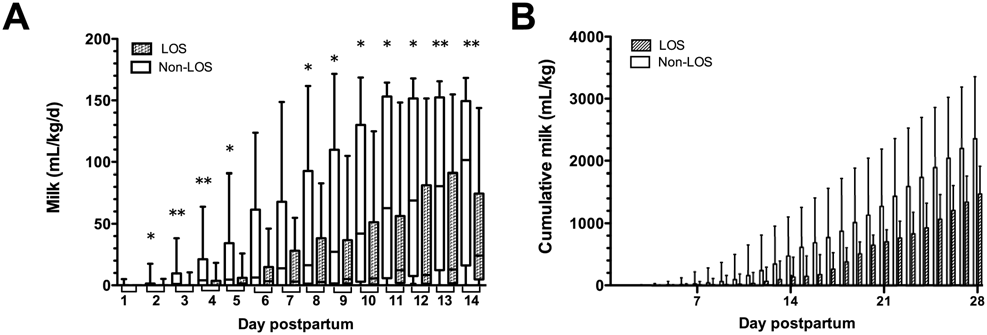
Consumption of breast milk (MOM+PDHM) by preterm infants in the case-control study. Boxplots show median and interquartile ranges of (A) daily breast milk consumption and (B) cumulative milk dose (mL/kg) consumed by preterm infants in the nested case-control study from days 1–14 and 1–28, respectively, showing non-LOS (clear boxes, n = 20) and LOS infants (shaded boxes, n = 20), comparing cases and controls using Wilcoxon matched pairs analysis. Symbols indicate *p<0.05; **p<0.01.

Several measures of breast milk consumption showed differing values between cases and controls (Table 3); both daily and cumulative doses of breast milk in non-LOS infants were significantly higher than LOS cases from day 2 postpartum onwards (Fig. 5) and, importantly, the non-LOS infants received significantly larger volumes of breast milk prior to LOS (first LOS case was diagnosed at day 7, median onset day 13 postpartum). Cumulative doses of breast milk achieved at day 28 were significantly lower in cases than controls (median 1,474 mL/kg and 2,356 mL/kg, respectively; p = 0.001).

**Figure 6:**
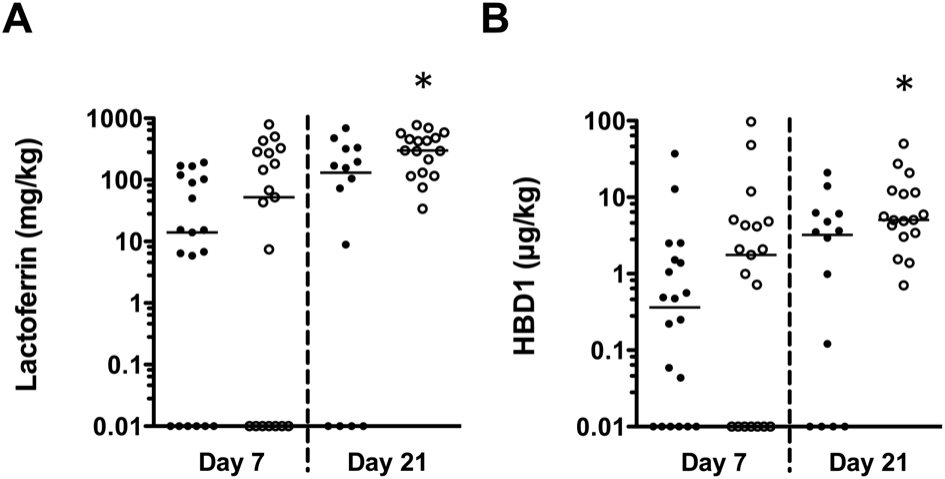
Consumption of AMPs at day 7 and 21 postpartum in case-control infants. Data show calculated consumed dose of (A) LF and (B) HBD1 in LOS infants (closed circles) or non-LOS infants (open circles) in the case-control study at day 7 (n = 19 each) and day 21 postpartum (n = 14 and n = 17, respectively), based on measured concentrations of AMPs in breast milk and reported milk consumption for each infant not consuming PDHM at the two time points on a log scale. Zero values calculated at day 7 (6 LOS, 7 non-LOS) and day 21 (4 LOS, zero non-LOS) were assigned a value of 0.01 for illustration purposes on the log scale. *p<0.05 comparing matched pairs.

The range of total daily LF consumed by infants ranged from 0–794 mg/kg and 0–94 μg/kg for HBD1 on days 7 and 21 postpartum (Fig. 6). The median doses of LF consumed by LOS cases were lower on day 7 (14 mg/kg LF in LOS cases and 52 mg/kg in controls, respectively; p = 0.30) and day 21 (131 mg/kg LF in LOS cases and 298 mg/kg LF in controls, respectively; p = 0.04). The median consumed dose of HBD1 at day 7 was 0.36 μg/kg HBD1 in LOS cases and 1.76 μg/kg in controls, respectively (p = 0.14), and 3.23 μg/kg and 5.03 μg/kg HBD1 were consumed by LOS cases and controls on day 21, respectively (p = 0.03).

## Discussion

Breast milk feeding decreases the incidence of LOS in preterm infants [7]. LOS pathogens may translocate from the gastrointestinal tract [3, 22], and therefore antimicrobial molecules found in breast milk could contribute to protective mechanisms through controlling bacterial load in the gastrointestinal tract, immune modulation, or alteration of the gastrointestinal microbiome. In this study, we found that concentrations of lactoferrin and defensins found in preterm milk are sufficient to cause inhibition of sepsis-causing bacteria and correlate with bacterial inhibition in milk. In a case-control study that compared matched infants with and without LOS, lower total consumption of breast milk, and hence of AMPs, was associated with LOS in preterm infants. Lactoferrin spiked into LBWF at the concentrations found in milk had similar bacteriostatic activity as whole milk, which supports its potential use as an intervention to prevent LOS.

The levels of AMPs in preterm breast milk measured were highly variable although, with the exception of LL-37, AMPs were detected in most breast milk samples. Both AMP levels and antibacterial activity were significantly higher in day 7 than in day 21 breast milk. Temporal changes to immune factors in breast milk have previously been reported [23] although, to our knowledge, this is the first report of temporal changes in antibacterial activity of breast milk. The reported ranges of LF concentrations in preterm breast milk are comparable to our findings [18, 19, 24]. Samples in this study had defensin values in the ranges described by others, but it is difficult to compare our data with the median values reported in milk in these studies due to variability in sampling time points and methodological differences between studies [25, 26]. The retention of breast milk AMPs measured in this study could have been affected by the opportunistic collection of breast milk stored in the neonatal unit, which included collecting frozen breast milk, and resulted in more than one freeze-thaw cycle prior to measurement of AMPs. Therefore, the values of AMPs reported in this study could be lower than if we had tested fresh milk. Storing milk at 4°C or -20°C and treatment with freeze-thaw cycles are common practices in our NICU, making the results biologically relevant for preterm infants in similar units. However, these data may not be generalisable to infants consuming fresh milk.

LF and its derivative peptides have well-described antibacterial activities [10, 27–29] yet, to our knowledge, no studies of LF activity based on actual breast milk concentrations have been previously performed. Spiking LBWF with average breast milk concentrations of LF results in inhibition of bacterial growth similar to that of breast milk, regardless of other factors that may differ between LBWF and milk. A methodological limitation of our spiking method was the relatively higher dilution of formula needed for the high and median concentrations of LF, due to insolubility of the LF at the concentration required to dilute the solution into formula. However, we did not find evidence of decreased growth of bacteria in control treatments containing formula diluted 20% by water in the experiments with ferric citrate (Fig. 4) compared to neat formula used in the formula spiking assays (Fig. 3), which suggests that growth factors in formula were not limiting at this higher dilution. Our data indicate that in many preterm breast milk samples, defensin peptides are not present in sufficient quantities to have significant antimicrobial activity. In samples where levels are sufficient for antibacterial activity to occur, the amount of antimicrobial activity is small relative to the activity of LF. Published data on defensins and cathelicidin antibacterial activities are often restricted to specific pH and osmolality conditions [30], with concentrations in the μg/mL range [31]. These conditions may occur in neutrophilic granules, but few of our cell-free breast milk samples had defensin levels in this range, and milk is reported to contain approximately 7–70 μM sodium [32], emphasising the need for biologically relevant activity assays. We did not investigate the mechanism of inhibition beyond addition of iron to lactoferrin during bacterial assays, and so cannot conclude whether the observed effects are related to bacterial binding or membrane effects, or if any clumping of bacteria still occurred after thorough mixing of samples. The direct effects of these molecules on bacteria could be examined in future research to identify specific effects on bacterial structure or function when exposed to AMPs in milk. Since the formula spiking antibacterial assays were based on the ELISA results, some of the defensins could have greater activity in fresh milk than observed in our experiments, due to higher concentrations. Regardless, any activity of other AMP molecules in breast milk may be redundant, since LF is sufficient at the average concentration found in milk to inhibit sepsis-causing microbes alone, at least *in vitro*. This applies to both molecules included in the analysis, as well as others that were not selected to be measured, such as lysozyme, which was not selected because both *S. epidermidis* and *S. aureus* display significant resistance to lysozyme activity [33], and the concentrations of lysozyme reported to inhibit the growth of bacteria (5.4 mg/mL [34] and 16 mg/mL [35]) are much higher than the levels reported in breast milk (240–370 μg/mL) [36]. If the levels of LF found in most frozen preterm milk samples sufficient to inhibit LOS-causing bacterial growth, then it is possible that it could be used alone as a supplement to modulate the gut microbiome or prevent LOS in preterm infants who do not consume enough breast milk LF.

The data derived from our formula-spiking experiments suggest that defensins display very little antibacterial activity compared to that of whole milk. Exposure of milk to proteases and reduction in the gut may result in more active forms being released during digestion [37]. Therefore, we cannot exclude the possibility that, following ingestion *in vivo*, the activity of the breast milk antimicrobial molecules may differ to the activity observed *in vitro*. There are no data on the effects of digestion of defensins or cathelicidin in infants from breast milk. In our formula-spiking experiments, recombinant forms of HBD1 and HBD2 used were representative of the active peptide and, therefore, this should not have affected the outcome of the spiking experiment in the absence of exposure to digestive enzymes. However, LF used in experiments was derived from undigested human milk and, although there is evidence that LF partially survives digestion, the process may release more active antibacterial peptide derivatives [38]. Breast milk contains trypsin and other proteases, so it is possible that the digestion process begins in milk prior to ingestion by the infant [39]. The HD5 used in our experiments was a 17 amino acid peptide from within the active peptide region. Although some activity was observed in spiking assays, we were unable to detect activity of HD5 against *E. coli* at 100 μg/mL according to the method of Porter *et al*., who had previously demonstrated activity against this organism in this range [40] and, therefore, it is possible that the activity of this HD5-derivative does not reflect the activity of the form of the peptide present in the gut. Despite this, given that the amounts of HD5 we detected were approximately 1,000-fold lower than the MICs reported for this molecule [41], it is unlikely that activity would have been observed at the concentration used for these experiments, regardless of the source of peptide. Our results reflect necessarily simplified experimental conditions compared to the complexity of the gastrointestinal tract, and further research will be required to disentangle the effects of specific AMPs on the gut in animal models or clinical studies.

This study confirms previous findings that breast milk has bacteriostatic effects against some LOS pathogens [12–14, 42], and extends previous research to show that growth of *S. epidermidis*, the most commonly isolated LOS pathogen, is inhibited by breast milk and breast milk-concentrations of LF, which can be reversed by exogenous ferric iron. Other investigators have reported significant effects of iron-containing milk fortifier on bacterial growth in similar assays [43], and Chan *et al*. [42] demonstrated that addition of iron to breast milk decreased or removed the antibacterial activity against *E. coli*, *S. agalactiae* and *S. aureus*. The antibacterial effects may be due to the ability of LF to sequester iron from the environment, preventing its use by bacteria for growth, demonstrated by Aguila *et al*. [44]. No infant in this study reached full feeds (150 mL/kg/d) in order to qualify for milk fortification before they developed LOS, and no milk samples used in experiments were fortified. These experiments reported the effects of ferric iron on LF antibacterial activity and, since iron supplements in infants are given as ferrous sulphate, the effect of such therapy on bacterial growth *in vivo* cannot necessarily be generalised or interpreted based on these findings. Therefore, whether functionality of breast milk LF or exogenous therapeutic bLF against LOS pathogens or sepsis outcomes are negatively affected by the concentrations of iron derived from milk, formula, or fortifier, requires further investigation.

We did not detect deficiencies in AMP concentrations or activities in the milk of preterm mothers whose infant developed LOS. However, at days 7 and 21 postpartum, there was an association between lower AMP doses consumed and LOS in preterm infants. Previous studies indicate that failure to establish full enteral feeds by day 14 (the median time of LOS onset) is a major risk factor for LOS [45]. In this cohort, infants who developed LOS did not commence enteral feeds until median day 5 postpartum, and on average did not reach full feeds until after 4 weeks postpartum, compared to median 19.5 days to full feeds in the control group. The reasons for delayed establishment of feeds are unclear, but may reflect a poorer clinical state in the first few days of life or presence of risk factors for feed intolerance, which were not recorded. To our knowledge, these are the first data to provide evidence of lower LF consumption in infants who develop LOS. Based on our results, LF may protect infants against LOS by regulating bacterial growth in the gastrointestinal tract, which could result in reduced translocation of bacteria to the bloodstream, as shown in animal models [46, 47], though other functions of LF such as immune modulation may play a role in protection [48]. Preliminary intervention studies using bovine lactoferrin (bLF) to prevent LOS in preterm infants indicate a possible therapeutic role [49–51], but the optimal dosing regime is unknown. Doses of 100 mg/d [49] and 200 mg/d [51] of bLF have been tested, but Manzoni *et al*. [49] reported that benefits in their study were only observed in infants whose birth weight was <1,000 g (those initially consuming the equivalent of >100 mg/kg/d). Our data support the need to adjust bLF doses for body weight. In addition, bLF doses may need to be increased, as we have demonstrated that the average amount of LF consumed by infants without LOS at day 21 was almost 300 mg/kg/d (with an upper limit of ~800 mg/kg/d). However, data from a preterm piglet model indicate that high doses of bLF can cause adverse outcomes [52], and it is unknown whether increased doses in humans would result in better outcomes, without adverse events. Further studies are needed to explore optimal dose and duration of LF supplementation in high-risk preterm infants.

The concentrations and activities of LF and defensins may be higher in colostrum than day 7 and 21 milk, which was not measured in this study. Despite this, earlier consumption of colostrum, which contains high concentrations of AMPs, does not appear to prevent LOS in very low birth weight infants [53]. All infants on this study consumed colostrum when enteral feeds were initiated. These data suggest that regular consumption of milk may be required to adequately protect infants during the greatest period of risk for infection. Given the evidence, future neonatal research may benefit from separating infants into ordinal categories of milk doses consumed over a relevant time period rather than more simplistic categories of formula-, donor milk- or breast milk-fed, since this study and the work of others have demonstrated the importance of milk dose in protection against LOS [7].

Antimicrobial proteins and peptides in breast milk may play an important role in the postpartum period in preventing LOS, possibly during gut colonisation, through mechanisms such as reducing bacterial overgrowth and translocation. In this study, the consumption of AMPs was significantly lower in preterm infants who subsequently developed LOS compared to matched controls. We have demonstrated that the concentrations of human LF and defensins are sufficient in stored preterm breast milk to inhibit the most significant sepsis-causing organisms *in vitro* and, furthermore, that LBWF containing physiological concentrations of human LF has similar ability as whole milk to suppress bacterial growth. This is the first evidence that these molecules are present in sufficient quantities in preterm milk to have effects on LOS pathogens’ growth, and suggests that one potential mechanism whereby breast milk and bovine LF interventions reduce LOS is by limiting bacterial growth in the gut. We found that prescribed milk consumption was significantly lower in preterm infants who developed LOS, highlighting the need for research to improve feeding tolerance. Interventions aimed at increasing breast milk consumption, or increasing the total quantity of milk AMPs consumed through supplementation (particularly LF), may reduce the risk of LOS in preterm infants.

## Supporting Information

S1 Fig Comparison of case and control infant mothers’ milk composition. Data show individual and median case and control breast milk concentrations on a log scale of: (A) LF; (B) HBD1; (C) HBD2; (D) HD5; (E) LL-37, and (F) protein, measured in breast milk using ELISA or BCA assay for protein at day 7 (n = 20 non-LOS, n = 20 LOS) and day 21 (n = 17 non-LOS, n = 14 LOS; 11 matched pairs) from individuals selected to participate in the nested case-control study, showing LOS cases (closed boxes) and non-LOS controls (inverted triangles).
(EPS)

S2 Fig Comparison of case and control breast milk showing colony-forming units of: (A) *S. epidermidis*; (B) *S. aureus*; (C) *E. coli*; or (D) *S. agalactiae*, after 4 h incubation in either infant formula (F), or day 7 and day 21 skimmed preterm breast milk samples from LOS cases (closed boxes) and non-LOS controls (crosses). The dashed line shows starting inoculum. Data show individual colony counts (CFU/mL), n = 16 for formula samples, n = 20 cases and n = 20 controls for day 7 breast milk samples and n = 14 cases and 17 controls for day 21 samples, on a log scale. A value of 10^3^ CFU/mL was assigned to samples where the colony count was below the limit of detection of the assay.
(EPS)
